# *red* - an R package to facilitate species red list assessments according to the IUCN criteria

**DOI:** 10.3897/BDJ.5.e20530

**Published:** 2017-10-19

**Authors:** Pedro Cardoso

**Affiliations:** 1 Finnish Museum of Natural History, University of Helsinki, Helsinki, Finland; 2 IUCN SSC Spider & Scorpion Specialist Group, Helsinki, Finland

**Keywords:** Area of Occupancy, Extent of Occurrence, extinction risk, International Union for the Conservation of Nature, red list index, species distribution modelling

## Abstract

The International Union for the Conservation of Nature Red List is the most useful database of species that are at risk of extinction worldwide, as it relies on a number of objective criteria and is now widely adopted. The R package *red* – IUCN Redlisting Tools - performs a number of spatial analyses based on either observed occurrences or estimated ranges. Functions include calculating Extent of Occurrence (EOO), Area of Occupancy (AOO), mapping species ranges, species distribution modelling using climate and land cover and calculating the Red List Index for groups of species. The package allows the calculation of confidence limits for all measures. Spatial data of species occurrences, environmental or land cover variables can be either given by the user or automatically extracted from several online databases. It outputs geographical range, elevation and country values, maps in several formats and vectorial data for visualization in Google Earth. Several examples are shown demonstrating the usefulness of the different methods. The *red* package constitutes an open platform for further development of new tools to facilitate red list assessments.

## Introduction

The IUCN Red List of Threatened Species is the most widely used information source on species extinction risk (Rodrigues et al. 2006, Mace et al. 2008), relying on a number of objective criteria (IUCN 2001). It has been used to raise the awareness of conservation problems and facilitate subsequent inclusion in lists of legally protected species, guide conservation efforts and funding, measure site irreplaceability and vulnerability, influence environmental policies and legislation and evaluate and monitor the state of biodiversity (Gardenfors et al. 2001, Rodrigues et al. 2006, Baillie et al. 2008, Mace et al. 2008, Martín-López et al. 2011, Juslén et al. 2016). The IUCN criteria are based on, among others, population size and decline, geographic range, fragmentation and the spatial extent of threats.

For the vast majority of species that lack appropriate data on population size and decline (Cardoso et al. 2011a), the most used criteria by far are B and D2 (Cardoso et al. 2011b, Lewis and Senior 2010), which are based on geographic range, namely the Extent of Occurrence (EOO), Area of Occupancy (AOO) and respective trends (see below for definitions). Yet, given the frequent incompleteness, bias and uncertainty of the geographical data (Akcakaya et al. 2000), many assessments end up being based on subjective expert opinion which reduces characteristics elemental to any scientific work such as the objectivity and reproducibility of the assigned risk category. There is an urgent need for researchers to deal with the Wallacean shortfall (the distribution of species is mostly unknown), i.e., by introducing easy-to-use tools to help in the assessments in the face of incomplete, biased or uncertain geographical data.

The IUCN Red List Index (RLI) (Butchart et al. 2004, Butchart et al. 2007) is a measure of overall changes in IUCN Red List status over time of a group of taxa. The RLI uses weighted scores based on the Red List status of each of the assessed species, giving us an indication of the overall trend of the group. The RLI approach helps to develop a better understanding of which taxa, regions or ecosystems (e.g. Juslén et al. 2016) are declining or improving. However, as yet no tools are available to calculate the RLI and its confidence limits.

Following a series of tools that have been recently developed to facilitate species assessments, such as a specific type of peer-reviewed article that directly feeds into the IUCN Red List (Cardoso et al. 2016), the R package *red* - IUCN Redlisting Tools (Cardoso 2017) was released in August 2016 and is being continually developed (now on its 10^th^ iteration) to tackle the above-mentioned shortcomings of existing options.

## Specification

The R package *red* performs a number of spatial analyses based on either observed occurrences or estimated ranges. Importantly, given the frequent shortcomings of available data, the package allows the calculation of confidence limits for EOO, AOO and the RLI. It outputs geographical range, elevation and country values, maps in several formats and vector data for visualization in Google Earth. The package is written in R and can be installed from the Comprehensive R Archive Network (https://CRAN.R-project.org/package=red). The newest, often a development version, is also available at GitHub (https://github.com/cardosopmb/red). The raw data accepted by most functions are: 1) a matrix of longitude and latitude or eastness and northness of species occurrence records, and 2) a raster* object as defined by the R package raster (Hijmans 2016) depicting either habitat or environmental variables of interest to the modelling of potential species distributions. The main functions are:

Extent of Occurrence (*eoo*) - Calculates the EOO of a species based on either records or predicted distribution. EOO is calculated as the minimum convex polygon covering all known or predicted sites for the species.

Area of Occupancy (*aoo*) - Calculates the AOO of a species based on either known records or predicted distribution. AOO is calculated as the area of all known or predicted cells for the species. The resolution is 2x2km as required by IUCN.

Recorded distribution of species (*map.points*) - Mapping of all cells where the species is known to occur. To be used if either information on the species is very scarce (and it is not possible to model the species distribution) or, on the contrary, complete (and there is no need to model the distribution).

Species distribution of habitat specialists (*map.habitat*) - Mapping of all habitat patches where the species is known to occur. In many cases a species has a very restricted habitat and we generally know where it occurs. In such cases using the distribution of the known habitat patches may be enough to map the species.

Species distribution modelling (*map.sdm*) - Prediction of potential species distributions using maximum entropy (maxent). Builds maxent models (Phillips et al. 2006, Elith et al. 2011) using function *maxent* from R package *dismo* (Hijmans et al. 2017). *dismo* requires the *MaxEnt* species distribution model software, a Java program that can be downloaded from http://biodiversityinformatics.amnh.org/open_source/maxent. Copy the file *maxent.jar* into the *java* folder of the *dismo* package. That is the folder returned by *system.file("java", package="dismo")*. When multiple runs are performed, it is possible to calculate confidence limits (two-sided) for the maps and consequently the EOO and AOO values. All cells predicted to be suitable for the species in at least 97.5% of the runs will be used to calculate the lower confidence limits. Conversely, all cells predicted to be suitable for the species in at least 2.5% of the runs will be used to calculate the upper confidence limits for EOO and AOO. Consensus maps are also calculated transforming the initial maps from probabilities to incidence, doing a weighted sum of these (each run is weighted as max(0, (AUC - 0.5)) ^ 2) and presence being predicted in the consensus map for cells with values > 0.5. There are numerous options within this function that should be carefully considered, please check the vignette of *map.sdm*.

Map of multiple species simultaneously (*map.easy*) - Single step for mapping multiple species distributions, with or without modeling. Outputs maps in asc, pdf and kml formats, plus a file with EOO, AOO and a list of countries where the species is predicted to be present.

Red List Index (*rli*) - Calculates the Red List Index (RLI) for a group of species. The IUCN Red List Index (RLI) (Butchart et al. 2004, Butchart et al. 2007) reflects overall changes in IUCN Red List status over time of a group of taxa. The RLI uses weight scores based on the Red List status of each of the assessed species. These scores range from 0 (Least Concern) to 5 (Extinct/Extinct in the Wild). Summing these scores across all species and relating them to the worst-case scenario, i.e. all species extinct, gives us an indication of how biodiversity is doing. Importantly, the RLI is based on true improvements or deteriorations in the status of species, i.e. genuine changes. It excludes category changes resulting from, e.g., new knowledge (Butchart et al. 2007). The RLI approach helps to develop a better understanding of which taxa, regions or ecosystems are declining or improving. Juslén et al. (2015) suggested the use of bootstrapping to search for statistical significance when comparing taxa or for trends in time of the index and this approach is here implemented. For each group, species are randomly sampled with replacement until the original number of species is attained. The confidence limits of the RLI values are the 2.5 and 97.5 percentiles of the runs. The change between dates is considered statistically significant if more than 95 % of the randomization values have the same sign (either increase or decrease) as the true values with no bootstrapping (Juslén et al. 2015, Juslén et al. 2016)

Red List Index for multiple groups (*rli.multi*) - Calculates the Red List Index (RLI) for multiple groups of species simultaneously.

Sampled Red List Index (*rli.sampled*) - Calculates accumulation curve of confidence limits in sampled RLI. In many groups it is not possible to assess all species due to huge diversity and/or lack of resources. In such case, the RLI is estimated from a randomly selected sample of species - the Sampled Red List Index (SRLI; Stuart et al. 2010). This function allows to calculate how many species are needed to reach a given maximum error of the SRLI around the true value of the RLI (with all species included) for future assessments of the group.

## Usage

I provide an example of a typical session using *red.* Start by loading the package and retrieving species records:

*library(red)*

*data(red.records)*

*spRec = red.records*

Ten unique records for *Hogna maderiana* (Walckenaer, 1837), a spider endemic to Madeira Island, have longitude and latitude data. Users can provide their own distribution data in either longitude/latitude (projected automatically to metric units) or any metric system such as UTM (in which case make sure that all data are in the same zone). One can calculate EOO and AOO (in km^2^) and extract information on the countries occupied:

*eoo(spRec)*

*aoo(spRec)*

*countries(spRec)*

For species whose records are known to be complete or at least depict their entire geographic range this is the basic information to be used in assessments. For further analyses a set of environmental layers are needed in raster format. These can be either: 1) user-provided, matching the species records data units, or 2) set up automatically by the package. The function *red.setup* will download worldclim (Fick and Hijmans 2017), elevation (Farr et al. 2007), and global land cover (Tuanmu and Jetz 2014) data at 30 arc-second and rescale them to 5 arc-minute resolutions (approx. 1 and 10 km respectively). The second resolution will be automatically chosen whenever the raw EOO (without modelling) is above 100000 km^2^ as it considerably speeds up the modelling and is much above the threshold of 20000 km^2^ for the category Vulnerable. For *H. maderiana* all data are available within *red*:

*data(red.layers)*

*spRaster <- red.layers*

*raster::plot(spRaster)*

One of these is the elevation for the region that can be used to extract information on species altitudinal range:

*altRaster <- spRaster[[3*]]

*raster::plot(altRaster)*

*points(spRec, pch = 19)*

*elevation(spRec, altRaster)*

**Figure 1.**
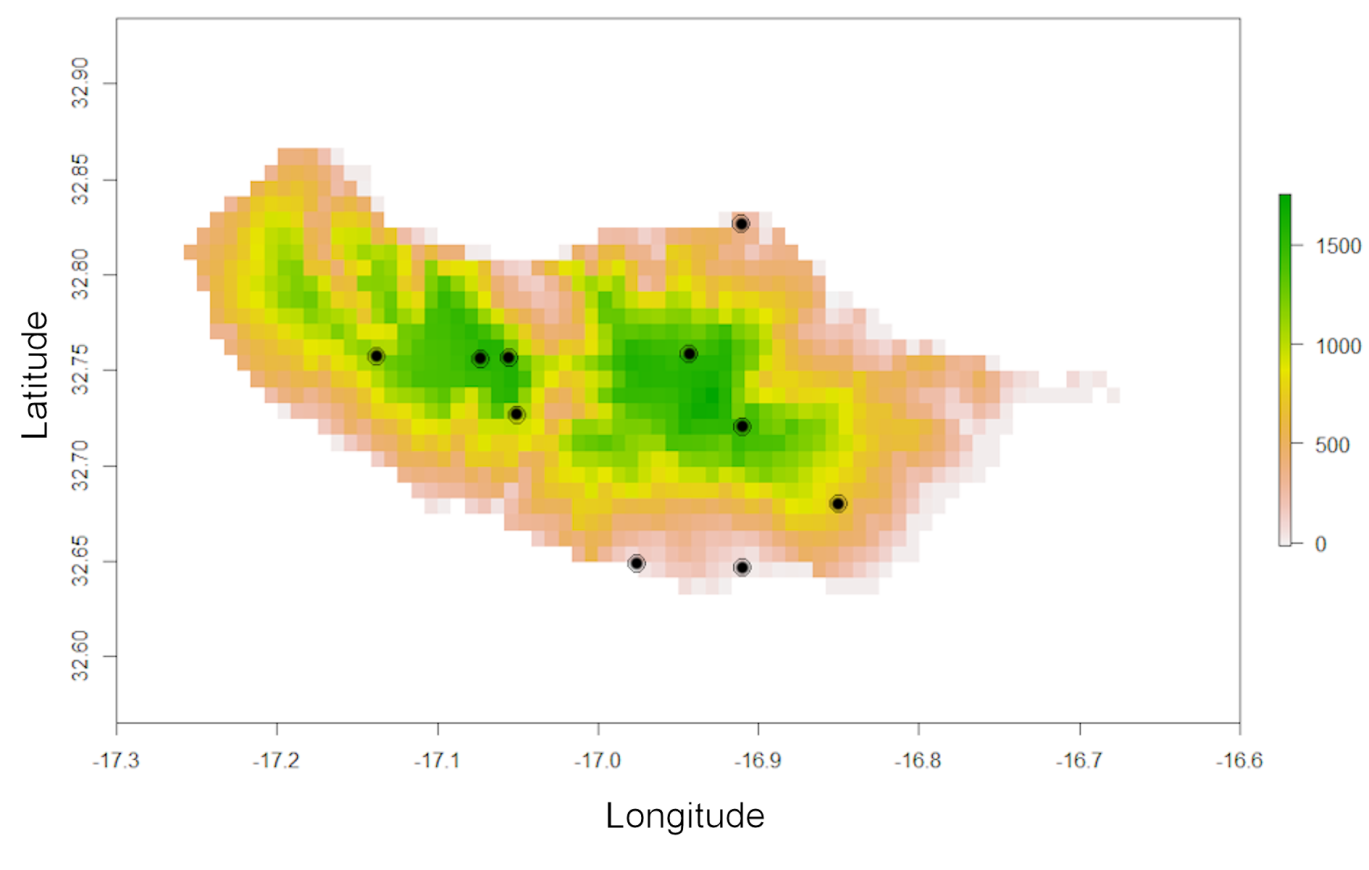
Elevation map (in meters) of Madeira Island with known records (circles) for the endemic spider species *Hogna maderiana* (Walckenaer, 1837). Environmental and species data are included in the *red* package.

Note that one record is in the sea (Fig. 1).

This may be due to spatial inaccuracy of the record or missing environmental data at the edges of the mainland or an island. In such cases, it might be good to move such points to the closest cell with environmental data:

*spRec = move(spRec, spRaster)*

*points(spRec, pch = 19, col=“red”)*

Unusual records can also be due to misidentifications, erroneous data sources or errors in transcriptions. These outliers can often be detected by looking at graphs of geographical or environmental space.

*outliers(spRec, spRaster)*

**Figure 2.**
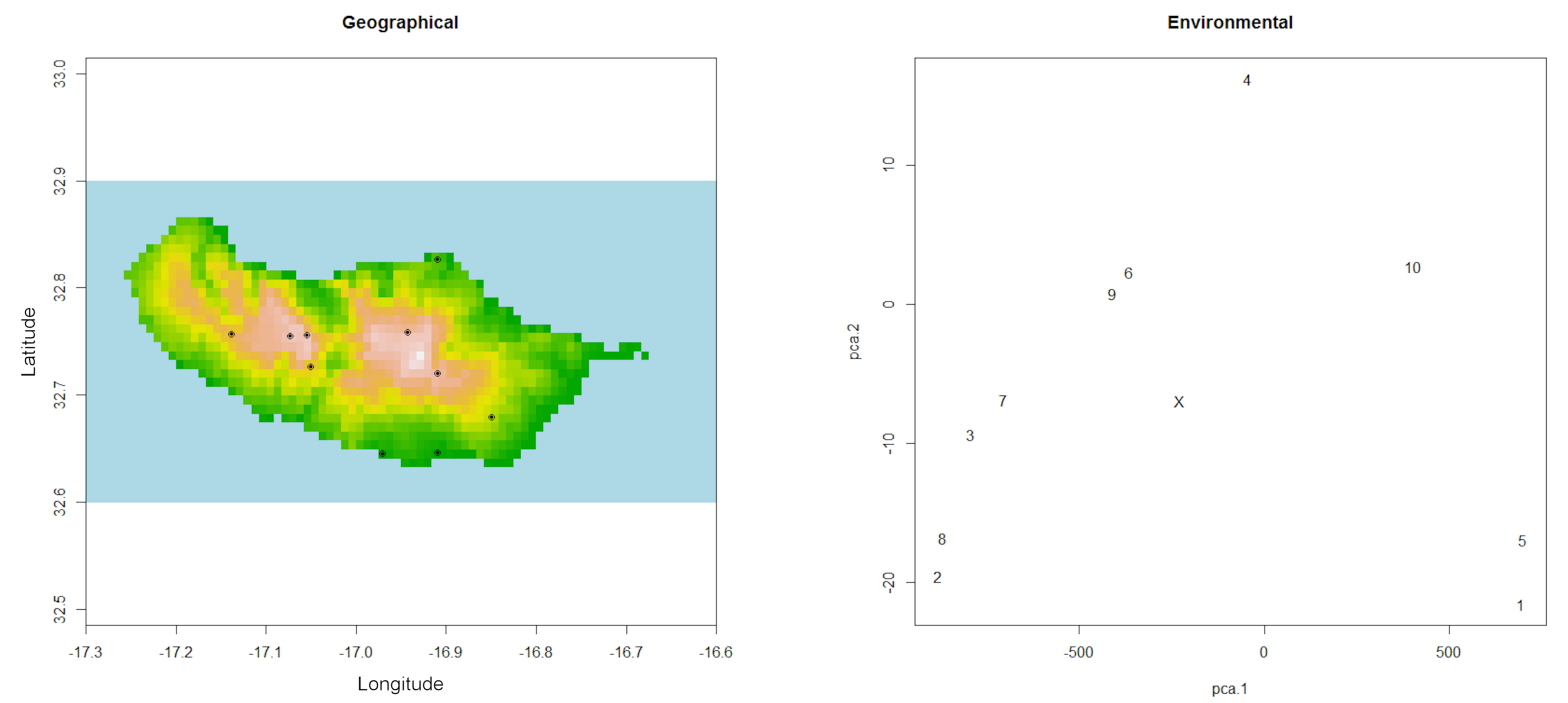
Output of the function *outliers*, depicting all sampling records in geographical and environmental space to facilitate detecting unusual records (X - centroid of PCA).

One might want to carefully look at records 1 and 5 (the ones at the southern coast of the island) as their environmental fingerprint is somewhat different from all other records as revealed by their distance to the centroid of the PCA (Fig. 2).

It is now possible to attempt mapping the species distribution:

*distrRaw <- map.points(spRec, spRaster)*

Note that the output is a list with a raster depicting all the cells where the species is recorded and a second element with the respective values for EOO and AOO.

*raster::plot(distrRaw[[1]])*

This map and values obviously assume that the records represent the entire range of the species. Yet, this is seldom true and in most cases some kind of extrapolation is justified. I propose two options within *red* to overcome the limitations of data. Often a species has a very restricted habitat and one generally knows where this habitat occurs. In such cases, using the distribution of the known habitat patches may be enough to accurately map the species. If one knows the spider species is currently restricted to forest areas:

*spHabitat <- spRaster[[4*]]

*spHabitat[spHabitat != 4] <- 0*

*spHabitat[spHabitat == 4] <- 1*

*par(mfrow=c(1,2))*

*raster::plot(spHabitat, legend = FALSE)*

*points(spRec, pch = 19)*

If one assumes the species does not currently occur outside forests that once occupied most of the island or in many of the small, isolated, forest patches, the following function retrieves only habitat patches known to be occupied:

*distrHabitat <- map.habitat(spRec, spHabitat, move = FALSE)*

*raster::plot(distrHabitat[[1]], legend = FALSE)*

**Figure 3.**
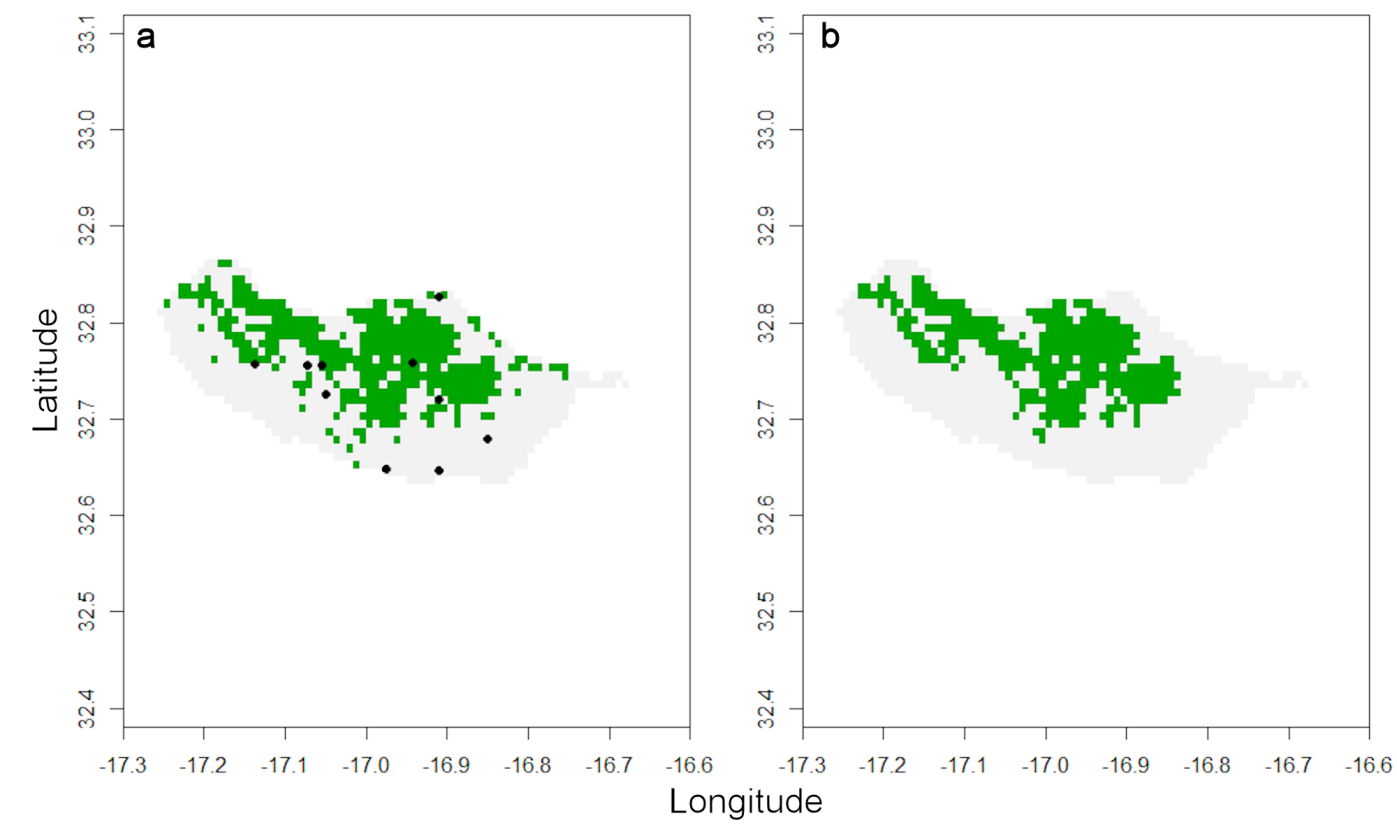
(a) Distribution of forest(ed) areas of Madeira (green) and historical records of *H. maderiana* (circles). (b) The species is restricted to the largest expanses of forest, highlighted with the function *map.habitat* (green).

The output is again a list with a raster depicting all the cells where the species is potentially present (Fig. 3) and a second element with the original and modelled values for EOO and AOO. It is also possible to move points outside forest areas to the closest cell with forest, in case these points do not represent subpopulations lost in the past (due to deforestation) but are due to georeferencing error, by setting parameter *move = TRUE*.

The second option available in *red* to overcome data deficiency is performing species distribution modelling (SDM) using Maxent (Phillips et al. 2006, Phillips et al. 2017). This technique however requires careful consideration of biases. SDMs are prone to spatial bias due to clumped distribution records derived from accessibility of sites, emphasis of sampling on certain areas in the past, etc. A thinning algorithm used in *red* eliminates records closer than a given distance to any other record. A number of random runs are made and the single run that keeps as many as possible of the original records is chosen (see also *Aiello-Lammens et al. 2015*):

*plot(spRec, pch = 19)*

*thinRec <- thin(spRec, 0.1)*

*plot(thinRec, pch = 19)*

**Figure 4.**
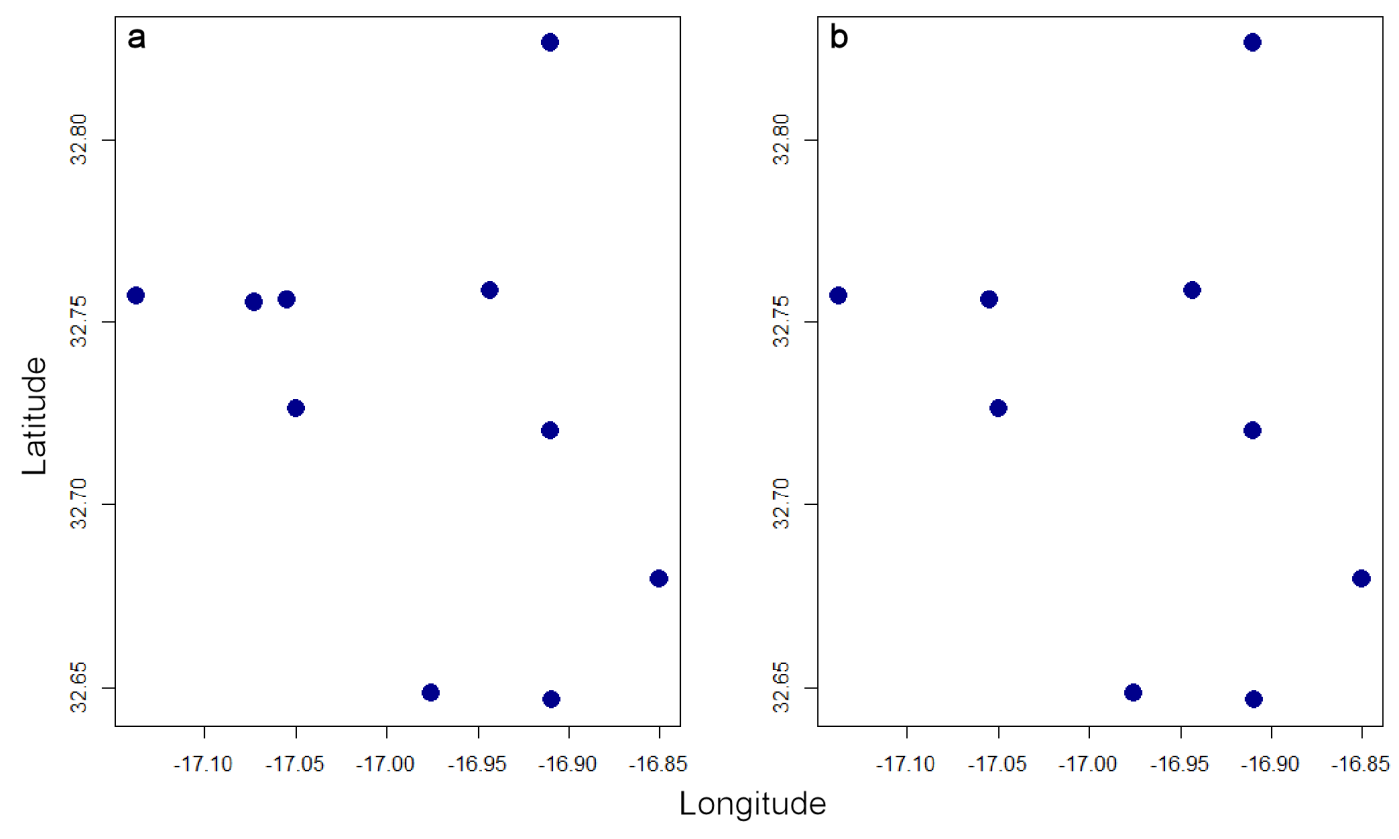
Original records of *H. maderiana* in geographical space (a) and the same records after spatial thinning using the function *thin* (b).

In this case, no records are closer than 10% of the maximum distance between any two after thinning (Fig. 4).

Overfitting is also a possibility in SDMs if the number of records is low compared to the number of predictor variables (environmental or other layers). The following function reduces the number of dimensions through either PCA or eliminating highly correlated layers:

*spRaster <- raster.reduce(red.layers[[1:3]], n = 2)*

*raster::plot(spRaster)*

**Figure 5.**
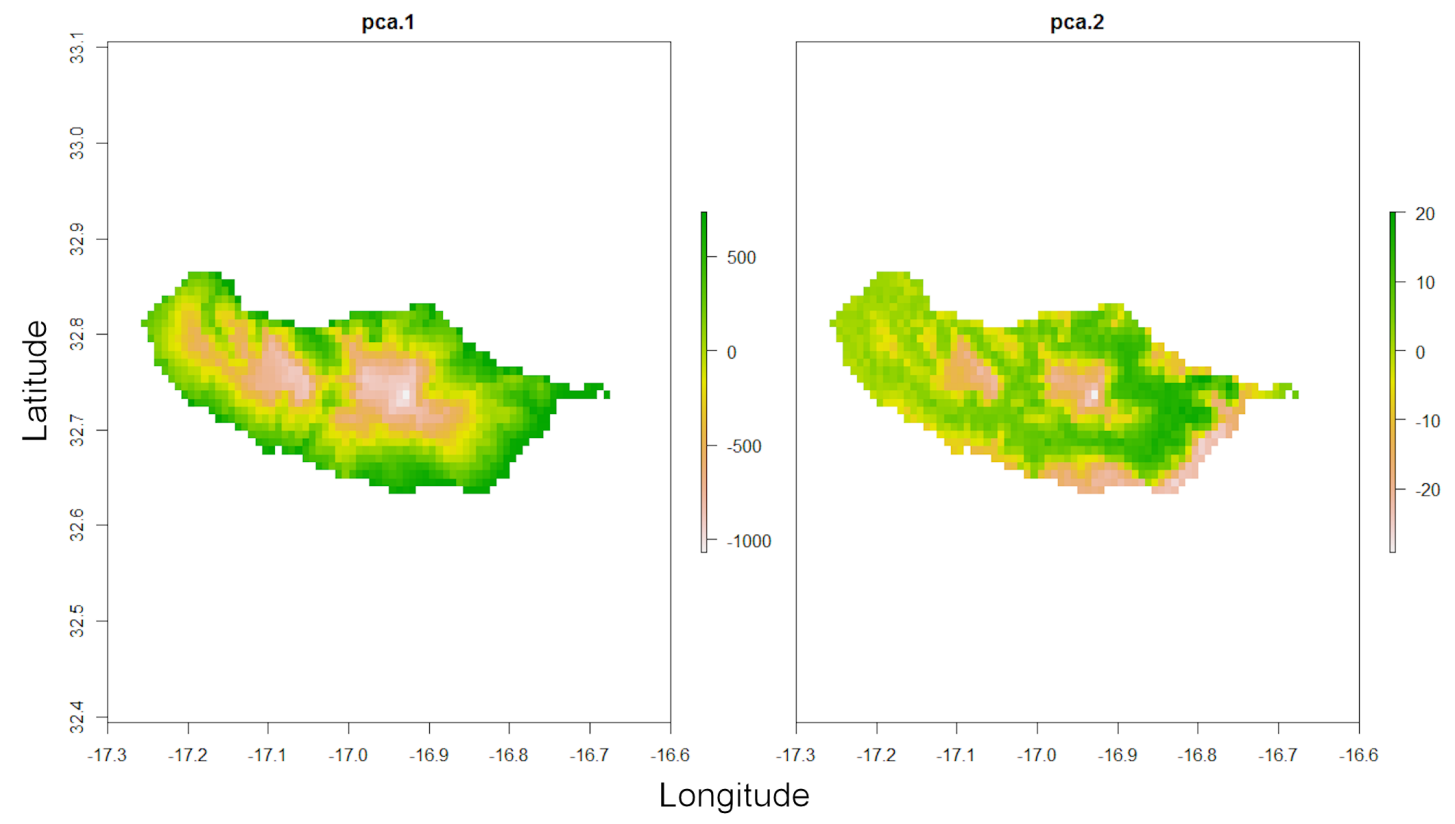
Result of the function *raster.reduce*, which decreases the number of environmental dimensions through PCA.

Note that land use was not used as it is a categorical variable (Fig. 5).

Only after all pre-processing of occurrence and climatic plus land use data is it possible to model the distribution. This example uses ensemble modelling (Araujo and New 2007) with 100 runs:

*spMap = map.sdm(spRec, spRaster, runs = 100)*

*raster::plot(spMap[[2]])*

*points(spRec, pch = 19)*

*raster::plot(spMap[[1]])*

**Figure 6.**
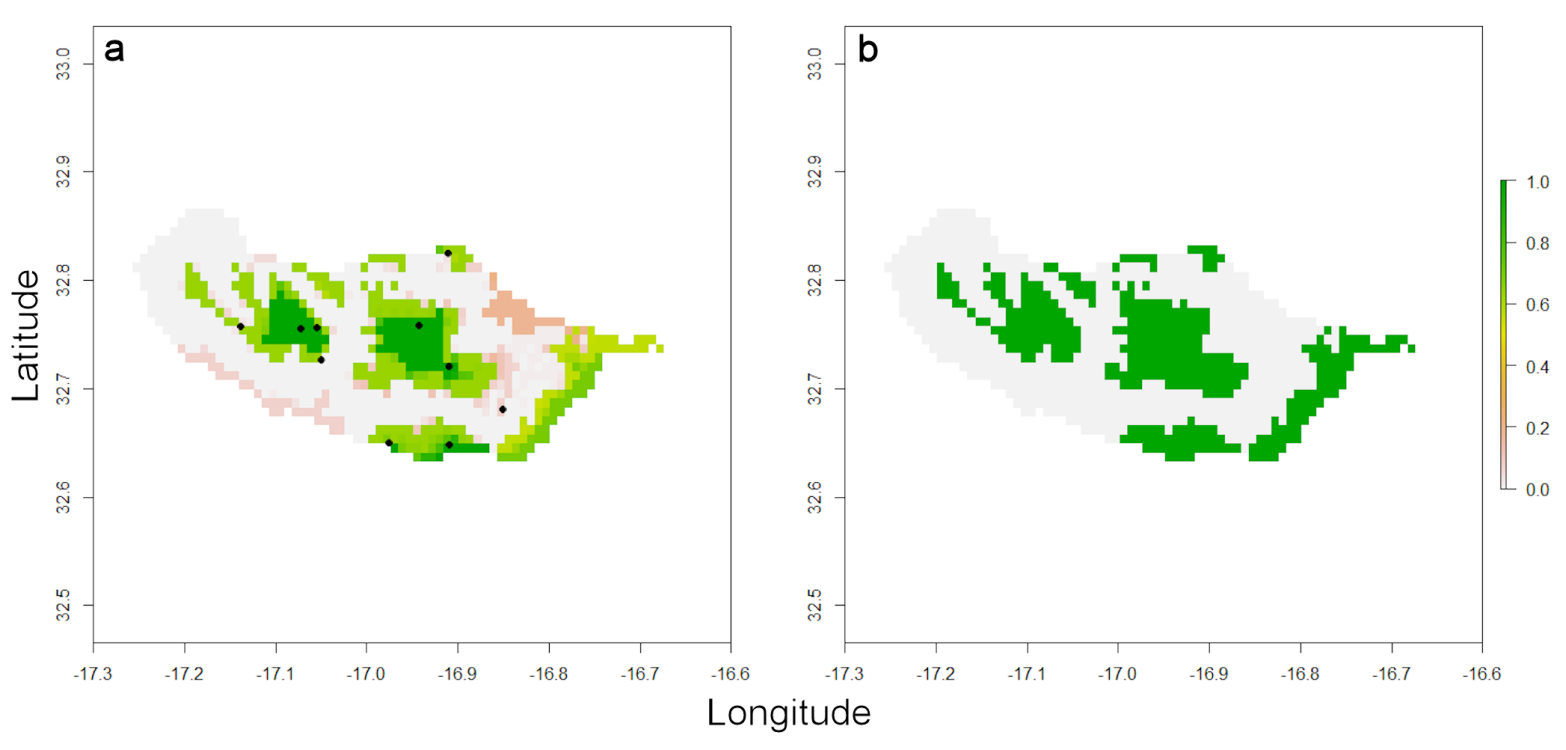
Result of ensemble modelling using the Maxent algorithm and the function *map.sdm*. The probabilistic map resulting from 100 runs (a) is converted to potential presence/absence using a consensus approach (b).

The consensus map (Fig. 6) reveals that the species could be present across the island.

IUCN requires maps to support the assessments, and *red* has the possibility of exporting them in several formats, namely kml:

*par(mfrow = c(1,1))*

*map.draw(spRec, spMap[[1]])*

*kml(spMap[[1]], filename = "spMap.kml")*

As an alternative or exploratory step, there is a function that performs most of this process in an automated way for multiple species (*map.easy*).

Finally, it is possible to calculate the Red List Index for single or multiple taxa simultaneously, including the calculation of confidence limits through bootstrapping:

*rliData <- matrix(c("LC","LC","EN","EN","EX","EX","LC","CR","CR","EX"), ncol = 2, byrow = TRUE)*

*colnames(rliData) <- c("2000", "2010")*

*rli(rliData)*

*rliData <- cbind(c("Arthropods","Arthropods","Birds","Birds","Birds"), rliData)*

*rli.multi(rliData, boot = TRUE)*

## Discussion

**Strengths**

The *red* package allows a fully data-driven and transparent assessment process within the most used statistical software package in ecological sciences - R. The software is open-source and being continuously developed (https://github.com/cardosopmb/red). It contains numerous tools, including methods not available in any other package or software. These include calculating EOO and AOO following the IUCN guidelines based on habitat or species distribution modelling, exporting maps in both pdf and kml formats, extracting information on elevation range and countries occupied by the species and calculating various formulations of the Red List Index. Importantly, it is the only software available to calculate all quantitative measures (EOO, AOO, RLI) with confidence limits, allowing the assessor to take data uncertainty into account.

**Caveats**

Although *red* was specifically designed to be as simple to use as possible, as most IUCN assessors have little familiarity with R or programming in general, minimum skills are still required. It is also still lacking functions for many of the criteria, such as estimating past or future changes in EOO, AOO or population size (but see future developments below).

**Alternatives**

To my knowledge, three other R packages intended to help with Red List threat assessments have been released. *rCAT* (Moat and Bachman 2017) calculates AOO and EOO from metric grids and includes a set of tools for coordinate conversion. *redlistr* (Lee and Murray 2017) calculates EOO and AOO from metric grids and their rates of decline. *ConR* (Dauby 2017) calculates EOO and AOO, outputs species maps and tries to estimate the number of subpopulations and locations. Both *rCAT* and *ConR* also try to estimate the IUCN categories from those data, although these would be only preliminary as more criteria beyond small range must be met, particularly for criterion B. A very useful online tool is *GeoCAT* (Bachman et al. 2011). Its main advantage is an excellent graphical user interface, which allows assessors to easily map and calculate EOO and AOO. Yet, *rCAT*, *redlistr*, *ConR* and *GeoCAT* lack most of the methods and flexibility found in *red.* Users should also be aware of the R package *rredlist* (Chamberlain 2017) which uses IUCN´s API to get information from the IUCN Red List, such as redlisted species per country or threats by taxon name.

**Future developments**

A number of developments are foreseen in *red*´s future. These include:

1. Calculating alpha-hulls for robust analysis of reductions or continuing declines in EOO (Burgman and Fox 2003).

2. Calculating the number of subpopulations and locations with a hint on fragmentation analysis.

3. Estimating past or future reductions in EOO and AOO based on dates of sampling records, land use/cover and climate change.

4. Incorporating phylogenetic and functional diversity information in RLI calculations.

5. Identifying candidate Key Biodiversity Areas (http://www.keybiodiversityareas.org).

The R package *red* tries to fill a gap in threat assessments according to the IUCN criteria using robust and replicable analytical methods. It is open source and any contributions to its continuous development are most welcome.

**Citation**

Researchers using *red* for publications should cite this article and in addition can also cite the *red* package directly. Updated citation information can be obtained by typing *citation("red")* in R.

## Web location (URIs) and repository

https://CRAN.R-project.org/package=red

https://github.com/cardosopmb/red
